# Mr. M'Fadzen on Water-Dressing in Wounds, Ulcers, Diseases of the Skin, &c.
*Ed. Med. and Surg Journ. No. 111.


**Published:** 1830-04-01

**Authors:** 


### Mr. M'Fadzen on Water-Dressing in Wounds, Ulcers, Diseases of the Skin, &c.*

The water doctors are oftentimes a byeword in the mouths of the profane and of those whose potations consist of a more exhilarating beverage, but they will now be supported by water surgeons. Dr. Macartney, of Dublin, taking a hint from Homer in the management of wounds, has revived the old water practice, and, as is usual with new medicines, or with old ones furbished afresh, it is achieving miracles. Far be it from us to hint that this water dressing is a milk and water matter; on the contrary, we bow, as in duty bound, to the majesty of potent facts. Mr. M'Fadzen, then, of Buttevant, in Scotland, having fortunately, met two of Dr. Macartney's pupils, and received intelligence of the worthy Professor's plan, resolved to adopt it in the dispensary of which he has the superintendence. Mr. M'F. is delighted to say, that "his success has considerably exceeded his most sanguine expectations, and he has little doubt that Dr. Macartney has effected, by scientific knowledge and acute observation, a valuable improvement in the art of practical surgery." So be it.

"The principle is to excite an agreeable sensation in the part affected; for if this be present inflammation cannot exist. The parts of which the human body is composed being naturally humid, the application of a fluid such as water, which is of the least irritating nature, affords an agreeable sensation, and prevents the necessity of inflammation, by doing away with the sense of injury sustained by the parts, it being a law in the animal economy that inflammation will not arise after an injury done to a part, unless that part feel sensible of the injury.

"The mode of applying this remedy is exceedingly simple, and fortunately attended with very little trouble. A piece of lint dipped in cold water is to be applied with the soft side to the part, and covered with oiled silk, which should extend considerably beyond the limits of the lint, and retained in its place by a light bandage, or any other means the practitioner may deem proper. Any other substance capable of preventing evaporation, and sufficiently light and pliable, such as very thin Indian rubber, would answer the purpose as well as oiled silk. The dressings should be removed three times a day, or less frequently if the secretions from the part are trifling, for the purpose of wetting the lint as it becomes dry, and freeing it from the secretions of the wound or skin, which would in a short time become irritant; therefore it is not sufficient that the lint should be merely moist, for this moisture may be occasioned by perspiration or other discharge of the part collected under

---

* Ed. Med. and Surg Journ. No. 111.

an impervious substance. Hence the lint must either be occasionally removed, or well-washed in cold water, and in like manner the oiled silk or Indian rubber.

"From what has been stated, it must appear that the good effects of this treatment depend on the production of steam at the temperature of the surface of the body, which, being retained by the impervious silk, subjects the part constantly to an atmosphere of that vapour.

"I hope it will not be considered irrelevant to mention here, that oiled silk is also a valuable substance for applying the emollient poultice, having this advantage over linen or calico, that it retains its moisture and heat, at least the heat of the surface over which it is placed, for a greater length of time. I subjoin a case to illustrate its use in this way, and to which I not only attribute the favourable termination of the case, but even the absorption of pus after fluctuation became evident."

With the foregoing rationale of the water-practice we will not quarrel, although we are very far indeed from agreeing in all the principles or theories advanced. Let us look at the *facts*, the cases, by which the general issue is to be tried.

CASE 1.—*Incised Wound.* A poor girl, æt. 16, presenting herself with contraction of the flexor tendon of the middle finger of the left hand, Mr. M'F. made a longitudinal incision down to the tendon in question, and cut it across. The finger was then straightened with some force, secured by a splint on the back of the hand, and cold water applied to the wound by means of the lint and oiled silk. The wound healed rapidly, and unless minutely examined, no cicatrix is observed.

CASE 2.—*Contused Wound.* M. B. æt. 10, had the nail of the great toe of the left foot shattered, and the integuments divided to a considerable extent, in consequence of a large stone falling on it. The parts were brought together and secured by sticking-plaster, and water-dressing applied over it. In a few days the dead and sloughy parts began to separate, and a bread and water poultice was substituted for the water dressing, but as soon as the separation was effected the latter was resumed. In about five weeks after the accident the cicatrization was far advanced, and the nail had considerably advanced in its growth.

Now with every disposition in the world to believe, and with as large a stock of faith as any medical journalist should possess, we really cannot discover any uncommon celerity in the foregoing cases, nor perceive any very miraculous effects from the cold water. We cannot but think, and we say it in all humility, that had any of the usual methods been adopted, the cases would have done just as well as they did under the water dressing of Dr. Macartney. Every body knows that it is usual, after dressing recent wounds, to apply spirit lotions, and it seems to us that the professors of the new light do nothing more than subtract the spirit and apply the water. In fact, the whole business reminds us strongly of the sympathetic powder of Sir Kenelm Digby, the virtues of which were so potent in healing a wound, when rubbed on the lance or the brand that had inflicted it!

But she has ta'en the broken lance,
And washed it from the clotted gore,
And salved the splinter o'er and o'er.

As a refrigerant lotion, or as a harmless application, cold water, applied in the manner directed, is well enough no doubt, but we do question much whether its virtues would go far in the treatment of an obstinate ulcer, or in any case that fairly required a decided remedy. When in London, Dr. Macartney paid a visit to St. George's Hospital, and at his suggestion and recommendation, some cases were treated by the water dressing. Sore and sorry we are to say, that its powers did not seem to us to be at all commensurate with the sanguine anticipations of its able patron, and, as the proof of the pudding is said to be in the eating, we may mention that this method is never pursued at present in the hospital. Had it been an elixir vitæ, probably it would not have sunk into so early an oblivion.

Before concluding, we beg leave to give Mr. M'Fadzen credit for singleness of purpose, and the greatest fairness in the statement of his facts. If we differ from him in opinion, it is perhaps to be considered as our misfortune, that we cannot see the truth so clearly as he does.

